# Efficacy and safety of weekly liquid alendronate in Korean postmenopausal women with osteoporosis: a 12-month, multi-center, randomized trial

**DOI:** 10.1007/s11657-024-01480-6

**Published:** 2024-11-26

**Authors:** Seungjin Baek, Seong Hee Ahn, Namki Hong, Da Hea Seo, Seongbin Hong, Yumie Rhee

**Affiliations:** 1 Department of Internal Medicine, Severance Hospital, Endocrine Research Institute, Yonsei University College of Medicine, 50-1 Yonsei-Ro, Seodaemun–gu Seoul, 03722 Republic of Korea; 2 Department of Endocrinology and Metabolism, Inha University Hospital, Inha University School of Medicine, Incheon, Korea

**Keywords:** Osteoporosis, Alendronate, Randomized controlled trial, Bone mineral density, Postmenopausal women

## Abstract

***Summary*:**

This study compared liquid and tablet forms of alendronate for osteoporosis treatment. After 12 months, both forms increased bone density to a similar degree with no significant differences in side effects. New low-volume liquid alendronate is as effective as tablets, offering an alternative treatment option for postmenopausal women with osteoporosis.

**Purpose/Introduction:**

Alendronate, despite its significant efficacy, poses challenges due to complex administration protocols and patient compliance issues, underscoring the need for various formulations. This study compared the efficacy and safety of once-weekly low-volume liquid alendronate sodium trihydrate (ALN-S), an oral solution, to once-weekly alendronate sodium (ALN-T), an oral tablet, in Korean postmenopausal women with osteoporosis.

**Methods:**

In a 12-month, multi-center, prospective, randomized, open-labeled, parallel trial conducted at two hospitals in Korea, 170 patients were randomized to alendronate solution (ALN-S) (*N* = 85) or alendronate tablet (ALN-T) (*N* = 85) groups. The bone mineral density (BMD) of the lumbar spine (LS), femoral neck (FN), and total hip (TH) was measured at baseline and after 12 months. Bone turnover markers (BTMs) were assessed at baseline, 6, and 12 months. The primary outcome was the percentage change in BMD of the LS, evaluated for non-inferiority.

**Results:**

After 12 months, both ALN-S and ALN-T groups exhibited a significant increase in LS, FN, and TH BMD, with no significant intergroup differences (ALN-S: LS 5.0 ± 0.6%, FN 1.8 ± 0.6%, TH 2.2 ± 0.5%; ALN-T: LS 5.2 ± 0.6%, FN 1.6 ± 0.6%, TH 1.8 ± 0.5%). ALN-S was found to be non-inferior to ALN-T for BMD change at LS (treatment difference: − 0.22%, 95% CI: − 1.84 to 1.40%), excluding the predefined non-inferiority margin of − 2.29%. Changes in BTMs did not differ significantly between groups. The frequency of adverse events was similar between groups.

**Conclusion:**

Liquid alendronate was non-inferior to tablet alendronate in increasing BMD in Korean postmenopausal women with osteoporosis, presenting a viable alternative when the tablet form is limited in various clinical scenarios.

**Clinical trial registration:**

The trial was registered with ClinicalTrials.gov (NCT05387200).

## Introduction

Osteoporosis is characterized by the systemic impairment of bone mass and microarchitecture, which results in fragility fractures [1]. Osteoporotic fractures are a major cause of morbidity and mortality, and pose a serious socioeconomic burden among older individuals [2].

Among the various medications available, bisphosphonates are commonly prescribed as a first-line treatment in patients with moderate-to-high fracture risk and as a follow-up after any anabolic or denosumab treatment in patients with very high fracture risk [3]. Alendronate, the most commonly prescribed and extensively studied bisphosphonate, reduces the risk of vertebral, nonvertebral, and hip fractures by 55%, 64%, and 47%, respectively [4].

Treatment adherence is a significant challenge, as recognized by the International Osteoporosis Foundation and European Calcified Tissue Society [5]. Over 50% of the patients discontinue oral bisphosphonate treatment within the first year [6]. The primary reasons for this discontinuation are drug-induced adverse effects and strict administration guidelines: the patient has to fast, swallow a large volume of plain water, and remain upright for 30 min [7].

To address this issue, several forms of alendronate have been developed. Effervescent-buffered soluble alendronate is more persistent and tolerable [8]. Oral jelly alendronate offers the same bone mineral density (BMD) gain with fewer upper gastrointestinal symptoms [9]. Liquid alendronate was initially formulated as 70 mg of alendronate in a 100 mL solution [10, 11]. Compared with tablet alendronate, liquid alendronate exhibits similar bioequivalence in terms of rate of urinary recovery (124.2 ug, SD [Standard Deviation] 147.3 ug) and maximum excretion rate (54.4 ug/mL, SD 50.3 ug/mL) as well as shorter and less variable transit times in both the standing and lying positions [12]. However, the efficacy of liquid alendronate remains uninvestigated.

Recently, 70 mg alendronate in a 20 mL solution with an additional 110 mL of water has been formulated, resulting in approximately 50% of volume reduction [13]. In this study, we aimed to evaluate the efficacy and safety of this newly developed drinkable alendronate in postmenopausal women with osteoporosis.

## Materials and methods

### Study design

**Fig. 1:**
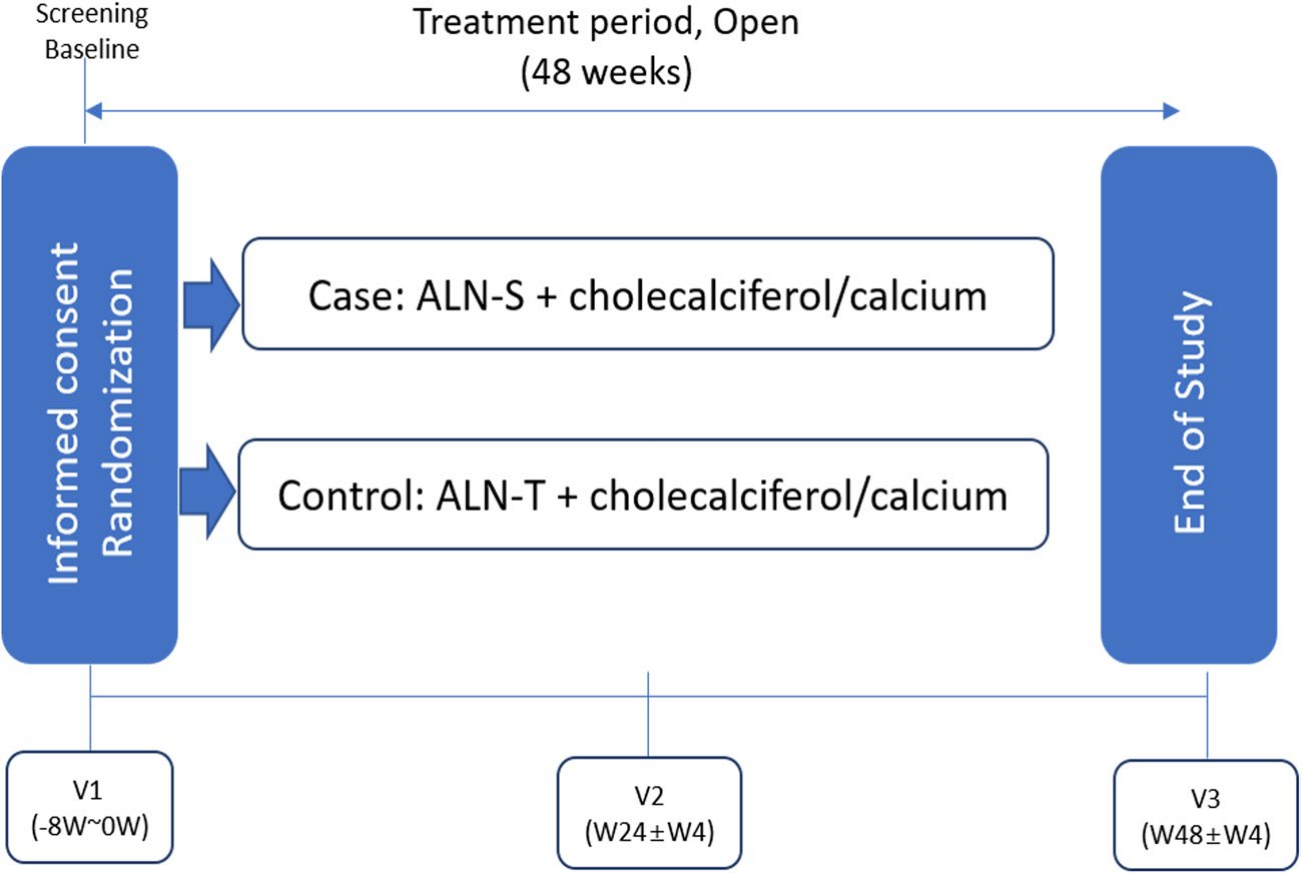
Study design. ALN-S, liquid alendronate sodium trihydrate; ALN-T, alendronate sodium oral tablet; BMD, bone mineral density; BTMs, bone turnover markers; C-telopeptide (CTX); procollagen type 1 amino-terminal propeptide (PINP)

This was a 12-month, multicenter, prospective, randomized, open-label, parallel trial conducted at two hospitals in South Korea (Severance Hospital and Inha University Hospital) to compare the efficacy and safety of alendronate solution and tablets. The patients were randomized into two groups: ALN-S (liquid alendronate sodium trihydrate, an oral solution, once weekly) and ALN-T (alendronate sodium, an oral tablet, once weekly). Both groups were administered daily supplements of 500 mg calcium and 1000 IU cholecalciferol (Fig. 1). The Yonsei University College of Medicine Ethics Committee and the Institutional Review Board and Ethics Committee of Inha University Hospital approved the study protocol (IRB No. 2021–01–018). The study design adhered to the ethical principles of the 1964 Declaration of Helsinki and its subsequent amendments. Written informed consent was obtained from all participants before enrolment, including protocol screening procedures and study drug administration.

### Study participants

We included postmenopausal women aged 50 years or older with BMD T-score less than − 2.5 at the lumbar spine (LS), femur neck (FN), and/or total hip (TH). Postmenopausal status was defined as the absence of menstruation for more than 48 weeks before screening with no other physiological or pathological causes of amenorrhea. Postmenopausal women with osteopenic BMD T-score (− 1.0 to − 2.5) and previous fracture history or high fracture risk in FRAX (major osteoporotic fracture [MOF] > 20% or hip fracture [HF] > 3% in 10 years) were also included. We excluded participants who had received any medication for osteoporosis within a year, had a history of bisphosphonate-related side effects (including medication-related osteonecrosis of the jaw, atypical femur fracture), risk of aspiration, severe renal or liver dysfunction, active malignancy, or secondary osteoporosis.

#### Procedures

BMD of the LS, FN, and TH was measured at baseline and after 12 months. BMD was measured using dual-energy X-ray absorptiometry (DXA) (Hologic Inc., MA, USA [Severance Hospital] or GE Lunar; WI, USA [Inha University Hospital]) at baseline and follow-up (12 months after the first treatment) in the LS, FN, and TH. The BMD T-scores were calculated according to the manufacturer’s reference range. Changes in BMD are expressed as mean ± standard deviation (SD) with percentage changes. Routine quality control of the bone density equipment was performed at each center according to the manufacturer’s protocol. The least significant change (LSC), calculated at the 95% confidence level of the coefficient of variation (CV), was 2.78%, 6.52%, and 3.27% for the LS, FN, and TH, respectively, at Severance Hospital, and 2.44%, 4.00%, and 3.44% for the LS, FN, and TH, respectively, at Inha University Hospital. The 10-year probabilities of MOF and HF were calculated using a Korea-specific computer-based algorithm (http://www.shef.ac.uk/FRAX).

#### Biochemistry

Blood samples were collected from the participants after they fasted overnight fast for at least 8 h. Serum levels of bone turnover markers (BTM), including C-telopeptide (CTX; Elecsys β-CrossLaps; Roche Diagnostics, Mannheim, Germany; intra-assay CV < 3.5%, inter-assay CV < 8.4% [Severance Hospital]; intra-assay CV < 6.0%, inter-assay < 7.4% [Inha University Hospital]) and procollagen type 1 amino-terminal propeptide (Elecsys total PINP; Roche Diagnostics; intra-assay CV < 3.6%, inter-assay CV < 3.9% [Severance Hospital]; intra-assay CV < 3.5%, inter-assay < 3.8% [Inha University Hospital]), were measured at baseline, 6 months, and 12 months.

### Outcomes

The primary outcome was the percentage change in bone mineral density of the lumbar spine (LSBMD) after 12 months, which was used to evaluate non-inferiority of liquid alendronate in relation to traditional alendronate tablets. The non-inferiority margin was selected as − 2.29%. Secondary outcomes included percent change in BMD at other sites (FN and TH); percent change in serum levels of BTMs (CTX, and PINP); compliance with treatment; and adverse effects. The non-inferiority margin for the difference in percent change from baseline in BMD between the treatment groups was based on the results of randomized controlled clinical trials comparing alendronate (10 mg daily or 70 mg weekly) with placebo in postmenopausal women with a follow-up period of at least 1 year and reported TH BMD data at 1 year [14]. To evaluate whether liquid alendronate retained at least 50% of the treatment effect of the alendronate tablet, non-inferiority margins were calculated as 50% of the lower bounds of the 95% CIs for the treatment differences (alendronate–placebo), which were based on a meta-analysis of random effect models [14]. The sample size was calculated to achieve 80% power, with a 10% dropout rate and a one-sided type I error rate of 2.5%. The standard deviation was assumed to be 5.0. With a non-inferiority margin of − 2.29, the derived sample size per group was 84 participants.

#### Treatment compliance and adverse events

Medication compliance is assessed by verifying the quantity of investigational product returned by study participants, which is then used to calculate the medication possession ratio (MPR). Treatment-emergent adverse events (TEAEs) refer to adverse reactions that did not exist before the administration of an investigational medicinal product but occurred after administration, or to adverse reactions that existed before the administration of the investigational medicinal product but worsened after administration. Adverse events (AEs) refer to any harmful and unintended sign (including abnormal laboratory results), symptom, or disease that occurs in a subject who has been administered an investigational medicinal product. Adverse drug reactions (ADRs) refer to any harmful and unintended reaction that occurs at any dose of the investigational medicinal product, where a causal relationship with the investigational medicinal product cannot be ruled out. Serious adverse events (SAEs) or serious adverse drug reactions (SADRs) refers to any adverse event or adverse drug reaction that occurs at any dose of the investigational medicinal product and meets one of the following criteria: results in death, hospitalization, persistent disability, congenital anomaly or otherwise considered medically important, such as the occurrence of drug dependency or abuse, blood disorders, or other medically significant conditions.

### Statistical analysis

The efficacy evaluation was based on a per-protocol analysis. The baseline characteristics of the study participants were analyzed using the two-sample *t*-test for numerical variables and the chi-squared test or Fisher’s exact test for categorical variables. Data are expressed as means ± standard deviation for numerical variables and as numbers with proportions for categorical variables. Changes in BMDs at 48 weeks compared to baseline in the same group were analyzed using a paired *t*-test. Analysis of covariance (ANCOVA), with baseline BMD as a covariate, was used to compare between-group differences in BMD changes at 48 weeks. The least squares (LS) mean difference between groups is presented with a 95% confidence interval. Regarding serum levels of BTMs (CTX and PINP), changes at 24 and 48 weeks were analyzed using a linear mixed model with the treatment group, time, and treatment group × time interaction as factors. Medication compliance at 24 and 48 weeks was assessed using Fisher’s exact test for categorical data and two-sample *t*-test for continuous data (MPR). Adverse events, including TEAEs, ADRs, SAEs, and SADRs were analyzed using the chi-square test or Fisher’s exact test. All adverse events were coded using the Medical Dictionary for Regulatory Activities (MedDRA) and categorized by system organ class (SOC) and Preferred Term (PT). All data were analyzed using IBM SPSS software ver. 26 or SAS software ver. 9.4. Statistical significance was set at *p* < 0.05.

## Results

### Baseline characteristics

**Fig. 2:**
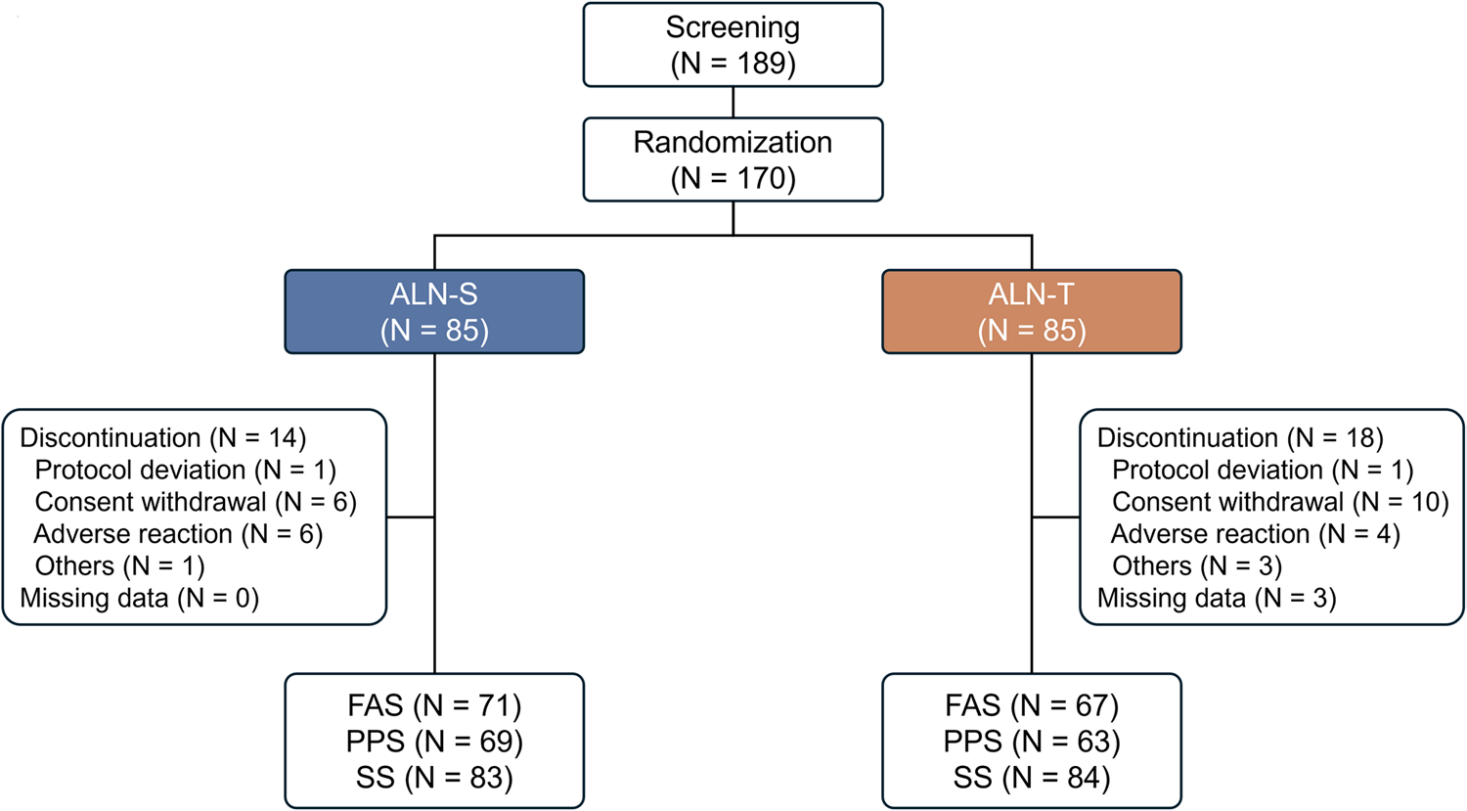
Flowchart detailing the categorization of participants. ALN-S, liquid alendronate sodium trihydrate; ALN-T, alendronate sodium oral tablet; FAS, full analysis set; PPS, per-protocol set; SS, safety set

A total of 189 participants were initially screened for this study. After screening, 170 participants were randomized into two groups of 85 participants each. Fourteen discontinuations occurred in the ALN-S group and 18 in the ALN-T group. The final analysis included 71 participants for the (FAS) and 69 for the (PPS) in the ALN-S group, whereas the ALN-T group included 67 and 63 participants for the FAS and PPS, respectively. The safety set (SS) analysis included 83 participants from the ALN-S group and 84 from the ALN-T group (Fig. 2). Table 1 presents the baseline characteristics of the two groups, which showed no significant differences in any of the measured parameters. The average age was approximately 64 years, and the mean LS T-score was approximately − 2.5.

**Table 1:** Baseline characteristics of the participants

	ALN-S (*N* = 69)		ALN-T (*N* = 63)		*p* value
	Mean (or *N*)	SD (or %)	Mean (or N)	SD (or %)	
Age (years)*	62.8	7.5	64.0	7.7	0.342
Height (cm)*	156.9	5.0	155.2	5.6	0.064
Weight (kg)*	55.4	7.7	55.5	7.2	0.923
Systolic blood pressure (mmHg)*	123.1	13.2	123.6	14.4	0.839
Diastolic blood pressure (mmHg)*	70.0	11.1	72.7	10.6	0.162
Calcium (mg/dL)*	9.3	0.3	9.3	0.3	0.633
Phosphorus (g/dL)*	3.8	0.5	3.7	0.4	0.763
Albumin (g/dL)*	4.5	0.3	4.5	0.2	0.749
ALP (U/L)*	74.8	21.3	68.4	17.3	0.060
BUN (mg/dL)*	15.1	4.0	15.8	4.2	0.305
Creatinine (mg/dL)*	0.7	0.1	0.7	0.1	0.875
Total cholesterol (mg/dL)*	183.7	52.0	186.7	31.2	0.683
Parathyroid hormone (pg/mL)*	40.8	22.5	38.7	13.6	0.518
25-hydroxy vitamin D (ng/mL)*	29.5	9.1	31.2	9.3	0.292
Baseline CTX (ng/mL)*	0.467	0.231	0.492	0.229	0.528
Baseline PINP (ng/mL)*	51.0	18.5	47.7	18.7	0.309
Baseline LSBMD (g/cm^2^)*	0.744	0.089	0.728	0.079	0.277
Baseline LS T-score*	− 2.4	0.7	− 2.5	0.7	0.526
Baseline FN BMD (g/ cm^2^)*	0.600	0.100	0.577	0.079	0.153
Baseline FN T-score*	− 2.1	0.7	− 2.2	0.6	0.295
Baseline TH BMD (g/ cm^2^)*	0.705	0.079	0.702	0.074	0.829
Baseline TH T-score*	− 1.4	0.6	− 1.4	0.6	0.693
FRAX: major osteoporotic fracture (%)*	10.3	5.2	11.4	5.4	0.224
FRAX: hip fracture (%)*	3.7	2.7	4.2	2.8	0.246
Previous fracture history^†^	21	30.4	20	31.7	0.871
Parents’ hip fracture history^†^	6	8.7	9	14.3	0.312
Current smoking^††^	1	1.4	2	3.2	0.606
Alcohol > 3 units/day	0	0.0	0	0.0	NA
History of glucocorticoid^††^	0	0.0	1	1.6	0.477
History of rheumatoid arthritis	0	0.0	0	0.0	NA
Secondary osteoporosis^††^	6	8.7	2	3.2	0.278

*ALN-S*, alendronate oral solution; *ALN-T*, alendronate oral tablet; *CTX*, C-telopeptide; *PINP*, procollagen type 1 N-terminal propeptide; *LS*, lumbar spine; *BMD*, bone mineral density; *FN*, femoral neck; *TH*, total hip; *FRAX*, fracture risk assessment tool

*Two-sample *t*-test, ^†^chi-square test, ^††^Fisher’s exact test

### Changes in BMD

**Fig. 3:**
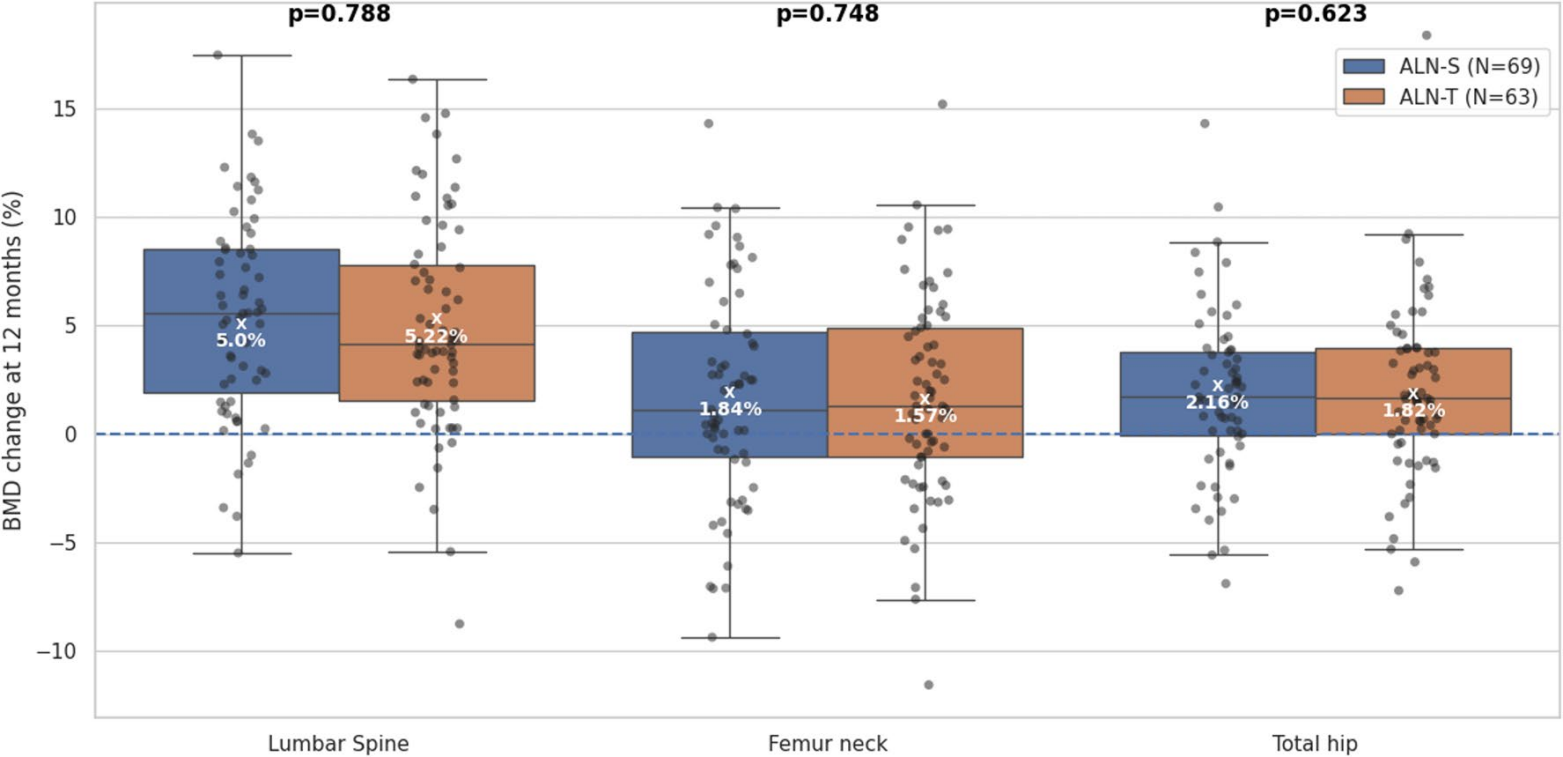
Changes in BMD. Boxplot shows bone mineral density (BMD) changes (%) at 48 weeks from baseline in lumbar spine, femur neck, and total hip with a line at median. A white “X” is marked at least square mean. The analysis of covariance (ANCOVA), with baseline BMD as a covariate, was used to compare between-group differences in the BMD changes for 48 weeks. ALN-S, liquid alendronate sodium trihydrate; ALN-T, alendronate sodium oral tablet

BMDs of LS, FN, and TH were measured after 12 months. The least squares mean of BMD change in the LS was not significantly different between the two groups: 5.0% and 5.2% gain was observed in ALN-S and ALN-T groups, respectively. BMD changes in the FN and TH were similar in ALN-S and ALN-T groups (FN, 1.8% vs. 1.6%; TH, 2.2% vs. 1.8%) (Fig. 3). Regarding LSBMD changes, liquid alendronate was non-inferior to alendronate tablets (treatment difference: − 0.22%, 95% CI: − 1.84 to 1.40%), excluding the predefined non-inferiority margin of − 2.29%. Although intergroup differences were not statistically significant, the differences from baseline were statistically significant in all groups and at all sites, which is consistent with the previously established efficacy of alendronate [14].

### Changes in serum BTMs

**Fig. 4:**
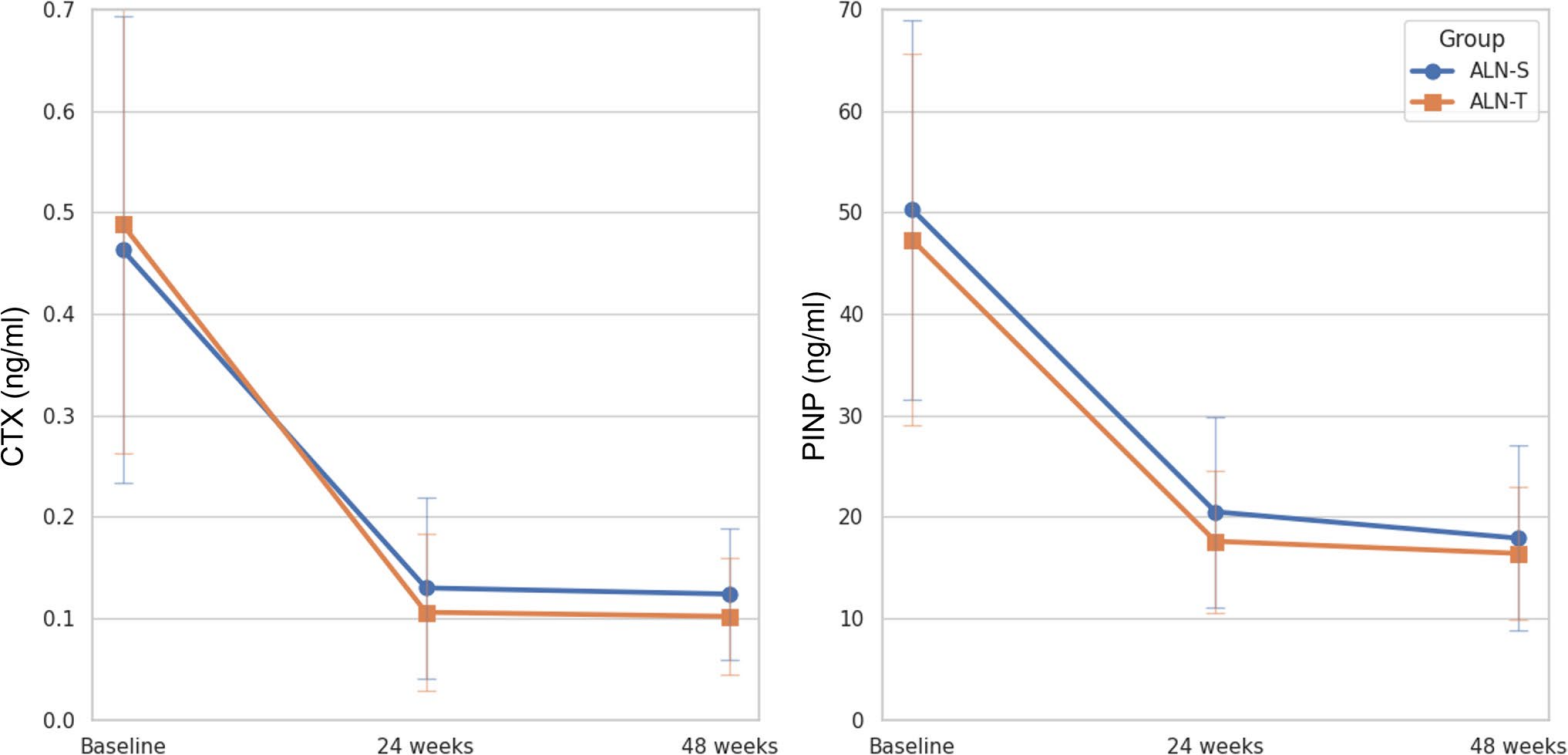
Percent changes in bone turnover markers. Mean and standard deviation are plotted as error bars. Changes at 24 and 48 weeks were analyzed using a linear mixed model. ALN-S, liquid alendronate sodium trihydrate; ALN-T, alendronate sodium oral tablet; BMD, Bone mineral density; BTMs, bone turnover markers; C-telopeptide (CTX); procollagen type 1 amino-terminal propeptide (PINP)

Reductions in serum levels of CTX and PINP were similar in both ALN-S and ALN-T groups (approximately 70% decrease in each group). Linear mixed model analysis showed a non-significant interaction between treatment type and time (*p* = 0.190 and *p* = 0.812, respectively), indicating that the two formulations (ALN-S and ALN-T) had similar effects on serum levels of CTX and PINP over time (Fig. 4).

### Treatment compliance rates

Treatment compliance was defined as MPR > 80%. Both ALN-S and ALN-T groups demonstrated high compliance at both time points; no statistically significant difference was observed between the two groups (Table 2). At 12 months, 100% of ALN-S participants and 98.4% of ALN-T participants were compliant (*p* = 0.477). Mean MPR at 12 months was 97.6 ± 2.8 and 98.1 ± 4.2 for ALN-S and ALN-T group, respectively (*p* = 0.401).

**Table 2:** Compliance rates of participants receiving alendronate solution and those receiving alendronate tablets

	ALN-S (*N* = 69)	ALN-T (*N* = 63)	*p* value
At 6 months			
Compliant*	69 (100.0)	62 (98.4)	0.477
MPR^†^	97.3 ± 3.8	97.8 ± 4.7	0.499
At 12 months			
Compliant*	69 (100.0)	62 (98.4)	0.477
MPR^†^	97.6 ± 2.8	98.1 ± 4.2	0.401

*ALN-S*, alendronate oral solution; *ALN-T*, alendronate oral tablet; *MPR*, medication possession ratio compliant; MPR > 80%

*N* (%), mean ± SD, *Fisher’s exact test, ^†^two-sample *t*-test

### Adverse events

The incidence of TEAEs was similar between the two groups, with 27.7% and 26.2% in ALN-S and ALN-T groups, respectively (*p* = 0.825) (Table 3). ADRs were observed in 13.3% and 7.1% of ALN-S and ALN-T participants, respectively (*p* = 0.192). Gastrointestinal disorders were the most common SOC-related adverse events, occurring in 9.6% and 8.3% of ALN-S and ALN-T participants, respectively (*p* = 0.768). No statistically significant difference was observed in the frequencies of adverse events. One case of Colle’s fracture and two cases of fractures (fibular and ankle) were observed in ALN-T and ALN-S group, respectively.

**Table 3:** Adverse events of two forms of alendronate

	ALN-S (*N* = 83)		ALN-T (*N* = 84)		*p* value
	*N*	%	*N*	%	
TEAE*	23	27.7	22	26.2	0.825
ADR*	11	13.3	6	7.1	0.192
SAE**	0	0.0	2	2.4	0.497
SADR	0	0.0	0	0.0	NA
GI disorders*	8	9.6	7	8.3	0.768
Cardiac disorders**	2	2.4	3	3.6	1.000
Musculoskeletal and connective tissue disorders**	7	8.4	2	2.4	0.099
Hepatobiliary disorder**	3	3.6	1	1.2	0.367
Infections and infestation**	4	4.8	6	7.1	0.746
Neoplasms benign, malignant, and unspecified**	0	0.0	1	1.2	1.000
General disorders and administration site conditions**	2	2.4	2	2.4	1.000
Nervous system disorders**	2	2.1	2	2.1	1.000
Skin and subcutaneous tissue disorders**	1	1.2	3	3.6	0.620
Respiratory, thoracic, and mediastinal disorders**	0	0.0	1	1.2	1.000
Renal and urinary disorders**	0	0.0	2	2.4	0.497
Endocrine disorders**	2	2.4	0	0.0	0.246
Psychiatric disorders**	0	0.0	1	1.2	1.000
Reproductive system and breast disorders**	0	0.0	1	1.2	1.000

*ALN-S*, alendronate oral solution; *ALN-T*, alendronate oral tablet; *TEAE*, treatment-emergent adverse event; *ADR*, adverse drug reaction; *SAE*, serious adverse event; *SADR*, serious adverse drug reaction; *chi-square test, **Fisher’s exact test

## Discussion

In this prospective, multicenter, randomized trial, we demonstrated that 12-month treatment with weekly alendronate solution is non-inferior to 12-month treatment with weekly alendronate tablet in increasing BMD at all sites in Korean postmenopausal women with osteoporosis. Furthermore, 12 months of weekly alendronate solution administration significantly suppressed serum BTMs in Korean postmenopausal women with osteoporosis.

Alendronate is routinely prescribed for the treatment of osteoporosis but is associated with high rates of discontinuation [6, 15]. Since poor adherence is associated with a smaller increase in BMD and higher fracture incidence, different alendronate formulations with potentially non-inferior efficacy and better tolerance are being developed [9, 12, 16]. Poor adherence rates are largely due to adverse effects and strict administration guidelines [7].

Alendronate solution demonstrated a shorter and more consistent gastric transit time regardless of whether the patient was standing or lying down [12]. Previous dosing requirements for the optimal use of bisphosphonates were rather strict: the patient was required to stay upright for at least 30 min and drink a large volume of water. Reduced variability in the gastric transit time could allow for more flexible and simplified dosing instructions, potentially improving patient adherence to treatment regimens. Although not yet approved, the ability of alendronate solution to be effectively absorbed in the supine position could provide a safer and more reliable treatment option for bedridden patients or those for whom following the postural requirements may be challenging.

Changes in water volume may also have a significant advantage. The alendronate tablet is administered with at least 200 mL of water. Alendronate solution required a similar regimen, wherein the patient was required to drink 100 mL of alendronate and an additional 230 mL of water. The newly formulated alendronate solution contains 70 mg of alendronate in a 20 mL solution and requires only 110 mL of additional water, which is approximately 50% of the volume previously required.

To the best of our knowledge, this is the first prospective randomized trial to investigate the effects of alendronate solution. Liquid alendronate provided the same BMD gain as the tablet form. The mean difference in LS BMD change was − 0.22% (95% CI − 1.84 to 1.40), which excluded the non-inferiority margin of − 2.29%, thus proving the non-inferiority of liquid alendronate compared to tablet alendronate. Similar results were observed for serum BTMs, with serum levels of both CTX and PINP suppressed by approximately 70% without significant differences. Although the efficacy evaluation was based on the per-protocol set, analyses performed on the full analysis set yielded similar results across all categories (data not shown). The results indicating that liquid alendronate increased the TH BMD by 2.2% are encouraging, as this suggests it effectively reduces fracture risk, according to the recent FNIH-ASBMR-SABRE study [17].

We also examined differences in compliance and adverse events. A previous retrospective study showed a large difference in the adherence to liquid alendronate and tablet alendronate [10]. Specifically, nearly 35% of 245 patients discontinued oral alendronate and 7.63% of 118 patients discontinued liquid alendronate after 12 months of therapy in a previous study, showing significant differences. Our study showed exceptionally good compliance for both forms of alendronate and thus did not show any significant difference, probably because of its prospective nature. Our results regarding adverse events were consistent with those of previous studies, showing no differences in the type and frequency of side effects between the two forms [12, 18]. A larger, well-designed study with higher statistical power and a longer follow-up period is needed to further investigate the differences between the two forms of alendronate.

The strengths of the present study include its randomized design and inclusion of a multicenter study population. Limitations include the open-label design, which may affect the reporting of AEs. In addition, although our study demonstrated the non-inferiority of liquid alendronate over 12 months, its findings may be limited by the study's power design, which targeted 80%, and an unexpectedly high dropout rate. Further research might be beneficial for assessing its long-term adherence, efficacy, and safety. Furthermore, qualitative studies exploring patient experiences with liquid alendronate, such as ease of administration, taste, and overall satisfaction, could provide valuable insights into its real-world acceptability and potential barriers to its use.

In conclusion, weekly liquid alendronate solution is safe and tolerable and can be a good option for postmenopausal osteoporosis patients who cannot tolerate the tablet formulation of bisphosphonates.

## Data Availability

The datasets used and analyzed during the study are available from the corresponding author on reasonable request.
